# Spiritual Care through the Lens of Portuguese Palliative Care Professionals: A Qualitative Thematic Analysis

**DOI:** 10.3390/bs14020134

**Published:** 2024-02-13

**Authors:** Juliana Matos, Ana Querido, Carlos Laranjeira

**Affiliations:** 1 Hospital Palliative Care Team, Local Health Unit of the Leiria Region, Hospital of Santo André, Rua das Olhalvas, 2410-197 Leiria, Portugal; juliana.matos@chleiria.min-saude.pt; 2 School of Health Sciences, Polytechnic University of Leiria, Campus 2, Morro do Lena, Alto do Vieiro, Apartado 4137, 2411-901 Leiria, Portugal; ana.querido@ipleiria.pt; 3 Centre for Innovative Care and Health Technology (ciTechCare), Polytechnic University of Leiria, Campus 5, Rua de Santo André—66–68, 2410-541 Leiria, Portugal; 4 Center for Health Technology and Services Research (CINTESIS), NursID, University of Porto, 4200-450 Porto, Portugal; 5 Comprehensive Health Research Centre (CHRC), University of Évora, 7000-801 Évora, Portugal

**Keywords:** spiritual care, qualitative study, palliative care, professionals, suffering, end of life, Portugal

## Abstract

Providing spiritual care is paramount to patient-centered care. Despite the growing body of data and its recognized importance in palliative care, spiritual care continues to be the least advanced and most overlooked aspect. This study aims to explore the perceptions and experiences of spiritual care from the perspective of PC professionals and identify their strategies to address spiritual care issues. Data were collected through semi-structured personal interviews and managed using WebQDA software (Universidade de Aveiro, Aveiro, Portugal). All data were analyzed using thematic content analysis, as recommended by Clark and Braun. The study included 15 palliative care professionals with a mean age of 38.51 [SD = 5.71] years. Most participants identified as lacking specific training in spiritual care. Thematic analysis spawned three main themes: (1) spiritual care as key to palliative care, (2) floating between “shadows” and “light” in providing spiritual care, and (3) strategies for competent and spiritual-centered care. Spiritual care was considered challenging by its very nature and given the individual, relational, and organizational constraints lived by professionals working in palliative care. With support from healthcare institutions, spiritual care can and should become a defining feature of the type, nature, and quality of palliative care provision. Care providers should be sensitive to spiritual needs and highly skilled and capable of an in-the-moment approach to respond to these needs. Further research on educating and training in spiritual care competence is a priority.

## 1. Introduction

Spiritual care is the cornerstone of whole-person palliative care (PC) [1,2], as initially emphasized by Dame Cicely Saunders through the concept of “total pain” [3], and later acknowledged by the World Health Organization’s definition of palliative care [4]. There is an increasing body of research indicating the significance of spiritual care throughout the latter stages of life. Professionals, especially in PC, often struggle with the suffering of the people in their care [5]. Problems and difficulties cause human suffering that calls into question the person’s integrity as a complex biopsychosocial and spiritual being [6]. Suffering is frequently experienced by end-of-life (EoL) patients and their families, during the continuous final stages of life, particularly the dying process, the death itself, and the subsequent mourning [7]. We assume that bodies do not suffer, but rather it is people as total beings who suffer [8]. Facing death can trigger existential anxiety in a person with a prolonged illness, which may evolve into existential despair, ultimately leading to spiritual suffering [9].

Evidence suggests that spirituality is a “dynamic dimension of human life that relates to the way persons (individual and community) experience, express and/or seek meaning, purpose and transcendence, and the way they connect to the moment, to self, to others, to nature, to the significant and/or the sacred” [10] (p. 2). Spirituality can alleviate suffering, promote healing, and provide meaning to patients and their families in their experience of dying [11,12,13,14,15]. This definition highlights the diverse nature of the spiritual domain and posits that ‘spirituality’ encompasses existential matters (such as inquiries into meaning, suffering, reconciliation, and hope), value-driven concerns (referring to dimensions that hold important significance for individuals), and religious tenets and reflections (about beliefs and actions) [16].

According to Nissen et al. [17], the process of spiritual care spans five sequential phases: (1) identifying spiritual needs and resources; (2) the meaning-making matrix (i.e., locating the ontological grounding of patients); (3) spiritual care treatment; (4) providing spiritual care; and (5) evaluation. Presenting spiritual care as a process implies the physical or psychosocial assessment of spiritual needs to screen for existential suffering or spiritual distress [18]. Viewing spiritual care as a process is particularly important since it emphasizes how transitioning from recognizing a patient’s spiritual needs to actually providing spiritual care requires intentional and thoughtful activities that consider the patient’s unique ontological foundation. It also requires identifying suitable experts to deliver spiritual care and help formulate an optimal spiritual care treatment plan. In this sense, care providers must be adequately prepared and responsible for addressing existential and spiritual aspects [9]. Spiritual care should be practiced by professionals to help people whose sense of meaning, purpose, and value is challenged by illness [9,19]. Spirituality and meaning-making are closely connected, implying that spirituality is a hermeneutic phenomenon only accessed through lived narratives [20]. Thus, spiritual care competence is vital for all healthcare professionals, as they must have the basic skills to assess and address the patient’s spiritual needs, especially in EoL when spiritual needs are greater [21,22]. Spiritual care should be based on compassion, support, empathy, and cooperation with other spiritual experts [5], allowing patients to express a desire for this form of care by their carers [23].

Studies have consistently shown that spiritual care has beneficial impacts on health-related quality of life, hope, and coping skills and can lower anxiety, depression, and suicide in patients [24,25,26,27,28,29], regardless of their age or medical condition. This includes patients with cancer, organ failure, and dementia, among others [30,31,32,33]. Furthermore, research indicates that a healthcare team’s failure to provide adequate spiritual support is linked to dissatisfaction with care, reduced use of hospice services, increased use of aggressive treatment, and higher costs [34,35,36]. Despite the growing body of data and its recognized importance in palliative care, spiritual care continues to be the least advanced and most overlooked aspect of palliative care [37,38]. Regardless of the need for competence in spiritual care, professionals feel inadequately prepared to implement spiritual care in their practice [15]. This difficulty has often been linked to a lack of understanding of the meaning of spirituality and spiritual care, as well as insufficient academic training concerning spiritual care [39,40].

Most studies on spiritual care in PC are from North Asia, America, and Western Europe [26,34]. Despite the vast available evidence, there is a notable scarcity of known studies from Southern European countries. Given their specific cultural, social, and religious context, studying this topic in this region is therefore important.

To the best of our knowledge, there is no qualitative research on spiritual care in palliative care conducted in the Portuguese context. This study uses a bottom-up approach to explore the perceptions and experiences of spiritual care from the perspective of PC professionals and identify their strategies to address spiritual care issues. In line with the research ambitions established by the Spiritual Care Taskforce of the EAPC [41], this study will help understand the European–Portuguese perspective on spiritual care in palliative care and also encourage professionals to attend to this dimension and provide more humanized care and quality spiritual assistance.

## 2. Materials and Methods

### 2.1. Study Design

This study was part of a larger multimethod project to examine spiritual care competence in palliative care [42]. The current study aims to comprehensively understand the intricacies of human experience within a natural setting, from the perspective of the individuals involved, following a qualitative descriptive using individual interviews [43]. Inspired by critical realism, we used a thematic analysis approach to analyze the collected empirical material. Critical realism captures the existence of different layers of reality and recognizes human fragility and that individual background and history can influence experience [44]. This is consistent with the antireductionist perspective that holds that social reality is in constant flux and motion and therefore its complexity should not be reduced to specific subsets of biophysical entities (as held by positivists) or discursive practices (as held by constructivists) [44].

The study followed the COnsolidated criteria for REporting Qualitative research (COREQ) checklist [45].

### 2.2. Study Setting, Participants, and Recruitment

Participants were recruited voluntarily from a palliative care unit of a tertiary hospital in the central region of Portugal. This unit offers person-centered palliative care services for patients suffering life-limiting illnesses, along with their families. The interprofessional collaborative approach targets pain and other biopsychosocial and spiritual symptoms.

A purposive sample was used to select participants. The inclusion criteria were as follows: (1) professionals who work in a palliative care inpatient unit and (2) provide direct care to patients and their families. The exclusion criteria were as follows: working in the unit for less than 6 months and being absent from work at the time of data collection, due to vacation or sick leave. The eligibility criteria were assessed by the main researcher (J.M.). No restrictions were imposed on age, sex, religious affiliation, or professional seniority, thus promoting maximum sample variation. In total, 15 professionals participated in the research until thematic data saturation.

### 2.3. Data Collection

Data were collected between September and October 2023 through individual semi-structured interviews in a private room in the palliative care unit. The interviews were conducted by a white female nurse (J.M.) with five years of professional experience in palliative care and under the supervision of a faculty member with experience in palliative care and qualitative research (C.L.).

Interviews began by collecting some personal data: age, sex, professional category, religious affiliation, years of professional experience, training in palliative care, and training in spiritual care. The semi-structured interview guide was prepared based on previous literature [46,47] and covered the following topics: (a) understanding of spirituality and spiritual care; (b) barriers and facilitators to spiritual care in palliative care practice; and (c) strategies used to implement spiritual care. To guarantee the validity of the interview guide and test its content and suitability for the study’s goals, the script was subjected to a pilot test by interviewing two palliative care experts. The interview questions were presented impartially, allowing individuals to provide their own viewpoints. The interviews were carried out in the participants’ native language (Portuguese). Field notes were taken during and after the interview. No repeat interviews were carried out. Interviews lasted an average of 30 min (varying between 20 and 60 min) and were digitally recorded and professionally transcribed verbatim.

The interviews were translated into English and coded with participant numbers (P1, P2, …) and professional area. During translation, textual quotations were initially translated word-for-word and subsequently adjusted to achieve equivalency in terms of meaning and interpretation. All authors perused both the Portuguese and English quotations.

### 2.4. Data Analysis and Trustworthiness

Inspired by critical realism, we performed a thematic content analysis based on the six steps established by Braun and Clarke [48]. Data familiarization was completed by thoroughly reviewing all transcripts. Relevant data were organized into comprehensible codes using WebQDA software (Universidade de Aveiro, Aveiro, Portugal). We coded samples of raw data (sentences, phrases, single words) that represented a certain meaning unit. Furthermore, the codes were classified into potential themes. Then, theme validity was confirmed by a meticulous examination of all codes and the whole data set. Themes were refined and named, resulting in the creation of a definitive thematic hierarchy. The final report was written with the assistance of a literature review [49].

Coding was conducted by two researchers: the main researcher (J.M.) coded all transcripts, while the second author with expertise in data processing (C.L.) independently co-coded them. When discrepancies or conflicts in coding emerged, the coders engaged in discussion until a consensus was reached. If disagreement persisted, a third reviewer was consulted to make the final decision (A.Q.). Approximately 30% of the data were double-coded, and inter-coder agreement was 90%.

To ensure trustworthiness in the analytic process, J.M. and C.L. had regular meetings to debate and defend the wording and content of codes, as well as the conceptual relationships and arrangement between codes, themes, and subthemes. Peer debriefing was employed to augment the credibility of the analysis. Following the well-known principles of qualitative research [50], we also maintained an audit trail consisting of field notes, coded transcripts, and comments and modifications from group coding meetings. The entire research team had experience in palliative care and was affiliated with Christianity. This naturally determined their personal perspectives on faith-based and spiritual practice. Notwithstanding, reference to the research team’s spiritual heritage was withheld throughout the study to prevent any indication the study was solely interested in favorable aspects of spiritual care.

### 2.5. Ethics

The study was carried out under the principles of the Declaration of Helsinki and after approval by the Ethics Committee of the Hospital Center of Leiria (70/CECHL/2023). All participants provided free and informed consent and the identity of each participant was safeguarded using an alphanumeric code. Participants were also informed that they could, at any stage of the study, choose not to participate. Respondents did not receive any monetary reward for joining the study.

## 3. Results

### 3.1. Sample Description

The fifteen participants were aged between 30 and 50 years (M = 38.51; SD = 5.71), with a majority of females (n = 14) (Table 1). Regarding the professional category, there were nine nurses, three physicians, two social workers, and one psychologist. Participants had an average of 13.6 years of professional experience and 3.3 years of work in palliative care. Although most practitioners had advanced training in PC (n = 9), only two participants had specific training in spiritual care (i.e., spiritual accompaniment retreat).

### 3.2. Qualitative Findings

**Figure 1:**
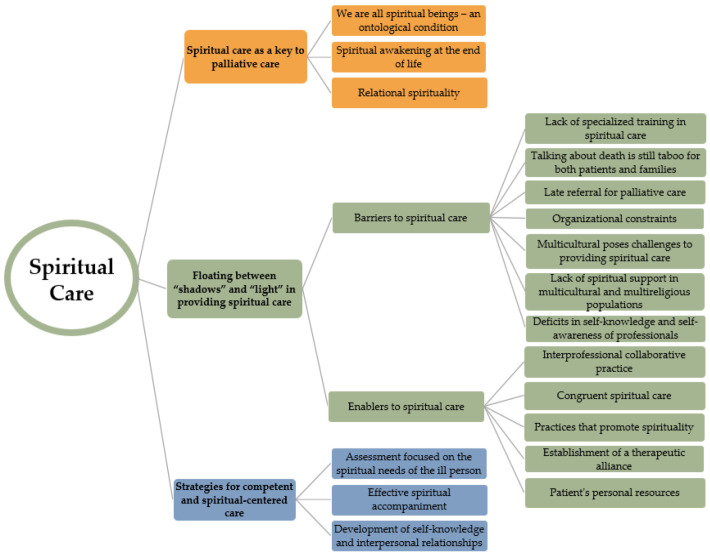
The coding tree of the thematic analysis.

Data from the interviews can be summarized in three main themes: (1) spiritual care as key to palliative care, (2) floating between “shadows” and “light” in providing spiritual care, and (3) strategies for competent and spiritual-centered care. Three subthemes were identified within the first theme: “We are all spiritual beings”—an ontological condition; spiritual awakening at the end of life; and relational spirituality. In the second theme, there were two subthemes: barriers and enablers of spiritual care. Lastly, in the third theme, we found three subthemes: assessment focused on the spiritual needs of the ill person; effective spiritual accompaniment; and development of self-knowledge and interpersonal relationships in work environments. All themes and subthemes are depicted in Figure 1. Each theme is described in turn, with illustrative quotes from the professionals’ data.

#### 3.2.1. Spiritual Care as Key to Palliative Care

Most participants understand spiritual care as essential to providing palliative care, mobilizing their knowledge, skills, and attitudes. Regarding the first theme, three subthemes were identified: (1) we are all spiritual beings—an ontological condition; (2) spiritual awakening at the end of life; (3) relational Spirituality.

(1) We are all spiritual beings—an ontological condition

Spirituality is an ontological condition of human beings. Some participants highlighted its universality as a less materialistic and more cosmic worldview. If we want to know ourselves as spiritual beings, we need to know our true essence, the stardust from which we are made. In this sense, spiritual assessment and care are a necessary pillar in PC.

*(P7; social worker): We are spiritual beings, and throughout our lives we think […] about our existence. Let’s look at our past, see our present, and let’s also think about our future. Therefore, spirituality is something that, in my opinion, is built throughout our lives and deeply determined by religious contexts.*

*(P4; nurse): Spirituality […], and having faith, are what characterize the person themselves, the essence of the person, and what defines them, in addition to the attributes they may have, physically, professionally, and socially.*

*(P6; psychologist): Spirituality is part of our life […], there is this need, a support, a basis for spiritual care at the end of life, in which there has to be a meaning for it or an attempt at peace in this terminal process.*

*(P11; nurse): I think it’s something beyond what we can see. […] it ends up being something very personal, something very individual.*

(2) Spiritual awakening at the end of life

Given the inevitability of death and the process of dying, patients and their families place great importance on spirituality, making it a key focus of their lives throughout this period. Spiritual awakening at EoL entails a transient amplification and deepening of consciousness, during which one’s condition of being, perception of the universe, and connection to it undergo a profound transformation, leading to a heightened sense of lucidity, revelation, and overall wellness.

*(P13; physician): The end of life is one of the most important phases to be worked on […] in palliative care. Indeed, it is at this stage of life that the person tries to find meaning in the illness and in the life and suffering they are experiencing. It’s about waking up, accepting the illness, and finding meaning for your life, your being, and your existence.*

*(P6; psychologist): Often this peace is found not only in the relationship with others, whether health professionals, family and friends, but in the transcendent relationship with a higher entity. When it is not named, when there is no particular name, they often tell me that they need to believe that there will be a higher entity that will guide their meaning in life; and which will certainly welcome them, they don’t know how, but they feel an awakening to the spiritual dimension.*

*(P7; social worker): Although spirituality is something that, in my opinion, is built throughout our lives, in palliative care it takes on another proportion, another dimension.*

(3) Relational spirituality

This subtheme pertains to spirituality in the context of social interconnectedness. Participants introduce the concept of relational spirituality to counteract an individualistic interpretation of spirituality. They believe that relationality is an essential component of existence and is expressed via social connections, especially in therapeutic relationships to achieve peace and spiritual well-being.

*(P7; social worker): […] Spiritual support for me […] is an extremely important dimension, because it will allow us to soothe the patient so that the family can be more soothed. We will allow, without a doubt, a calmer death […]. And I think it is our obligation as palliative care professionals, we also have this responsibility to know that this person can die in peace. It is our responsibility to help them in this task of pacifying themselves, no matter who they are […] so that when the time of death actually arrives, it will be as calm and serene as possible so that this life has not been lived in vain.*

*(P6; psychologist): The issue of spirituality goes hand in hand with the work we do towards reconciliation, pacifying families with the patient, the patient with themselves, and resolving other issues that concern that particular person, which provides openness to think about spiritual issues and to surrender to this more humanistic and self-loving side.*

*(P5; physician): I think that spirituality is very important in palliative care because it is an important determinant of the patient’s well-being and the patient’s interaction with health professionals and even interferes with the results that we can have in terms of global control of symptoms, in addition to the spiritual well-being itself.*

#### 3.2.2. Floating between “Shadows” and “Light” in Providing Spiritual Care

Professionals in PC acknowledge the significance of spiritual care, but often it is not incorporated into clinical practice. Participants highlighted several barriers and enablers to offering spiritual care in the palliative care context.

##### Barriers to Spiritual Care

Some “shadows” hinder professionals’ ability to provide spiritual care support. One of the main barriers highlighted by participants corresponds to the lack of specialized training in spiritual care, which limits their capacity to respond to the complexity of situations they face daily. As stated,

*(P7; Social worker): If I had specific training in spiritual care, it would help me approach situations differently and give me other skills. Without a doubt, it would be important.*

*(P4; nurse): I think it is very important for people to know because it is very difficult for us to experience what others are experiencing during the terminal phase. But I think that training in the area of spirituality is very important to become more sensitive to it […], and in this way we can help.*

*(P3; nurse): Training is what makes all the difference. I think that training makes a difference, even though I don’t have any and there isn’t training of this type. But I believe it enables us to manage all these emotions and deal with issues of transcendence.*

*(P5; physician): Basically, doctors and nurses are more trained to control symptoms than to deal with existential issues […]; it is extremely important to have training in spiritual care. It is an area that must be transversal to all health professionals, regardless of their area, so that we can all as a team identify the spiritual needs of the patient and be able to help in the best way or promote the patient to improve their spirituality.*

Two participants added that talking about death is still taboo for both patients and families, which makes approaching the spiritual dimension even more difficult.

*(P1; physician): Talking about death is something that families don’t try to do. There are few patients or families who have the time or the openness to talk about these things.*

*(P2; nurse): Families often don’t realize that it’s not just a symptomatic lack of control, and that’s why they end up not giving importance to the moments when professionals are supporting existential suffering. They prefer not to talk about the subject.*

*(P3; nurse): Some families sometimes have different opinions than the patient and others are also not so receptive to our presence or even interfere with the patient’s wishes.*

In organizational terms, the late referral for palliative care is another barrier to providing spiritual care. This concern often prevents patients from reconciling, seeing their wishes fulfilled, working on their legacy, and feeling connected with their own transcendence (i.e., developing transpersonal spirituality).

*(P13; physician): Many times we are unable to provide adequate spiritual support as referrals are late […]; they arrive late.*

*(P1; physician): Many patients come here in the last hours/days of their lives […], so we often cannot understand what the patient wants, their spiritual needs, how we can help them to calm down with themselves and others.*

Other organizational constraints such as staff shortages, lack of standards in professional-to-patient ratios, lack of time, and high workloads also configure variables that hinder professionals’ ability to respond. As stated,

*(P12; social worker): When we have a patient, we must be focused on them, but it is not easy to monitor so many people… The feeling I have is that I am always against the clock […].*

*(P4; nurse): We should have more time for spiritual care, but that doesn’t happen due to a lack of resources. We do our best!*

*(P7; social worker): The biggest barrier? Time, time. Literally, time. Because for spiritual care you need time. On the other hand, having a higher ratio of professionals in these palliative teams would help a lot.*

Spiritual care is an essential aspect of healthcare that is often overlooked, especially in a multicultural environment. Multiculturalism poses challenges to providing spiritual care, appearing as a barrier. According to participants, the flux of people from various cultures and religions has grown, so providing appropriate care requires understanding the diverse spiritual beliefs of patients.

*(P1; physician): We have had many immigrants, people who come to our country and who belong to Muslim, Ismaili, or Hindu communities, and this represents a challenge for palliative care teams as we do not have references in the community who can help us.*

*(P10; nurse): A reality that is changing in Portugal is more multiculturalism. Because there is another type of spiritual need you are not so familiar with, I feel that it is a difficulty.*

Likewise, the lack of spiritual support in multicultural and multireligious populations is a challenge for health professionals. The growing variety of patients raises concerns about the professional competencies and responsibilities related to addressing their religious, spiritual, and existential demands.

*(P12; Social worker): When we have people of other faiths […], who do we turn to? Apart from the chaplain, we have no one here who can help us. For example, if the patient wanted to speak to the Imam, we would have no way of offering this spiritual service.*

*(P6; Psychologist): Portugal is a country with a Catholic tradition, which means that we only have a priest as a member of the team. It is not easy to have a spiritual assistant available for each religious belief or confession. We end up, whether we like it or not, relying solely on the support of the Catholic priest.*

At the individual level, participants mentioned that deficits in self-knowledge and self-awareness of professionals regarding spirituality impact the quality of care. When engaging with personal spirituality and delivering sensitive spiritual care, the essential factors include having an open mind, being self-aware, and practicing adequate self-care.

*(P8; nurse): We all have personal difficulties, we are human, and we are not well every day, so despite trying to abstract myself, I assume that these difficulties could act as an obstacle in satisfying the spiritual needs of our patients.*

*(P13; physician): The problem lies in the poor self-knowledge of professionals. As a spiritual being, I am still making my way and sometimes I don’t feel comfortable bringing up the subject of spirituality because I still haven’t found all my answers […]; this requires us to do a lot of introspection and work ourselves, which is not always easy.*

*(P12; social worker): My main barrier is […] I don’t have spirituality refined within me. And therefore, I confess that I try to avoid this issue a little. If the question is objective, I tend to answer, if it’s not objective, I confess that I don’t raise the topic either, or I don’t ask many questions about the topic. […] Because the feeling I have is that if I do it, it will sound false or unprofessional because I don’t know how much I believe it, and so I don’t want to invent either.*

##### Enablers to Spiritual Care

As participants embark on the spiritual care journey, they connect with this trajectory’s “light”, using the resources they have to face all daily difficulties. One of the main facilitators corresponds to interprofessional collaborative practice. Effective interprofessional teamwork increases response capacity in more complex situations, promoting the quality of spiritual care.

*(P12; social worker): I’m lucky to work in a place where we work as a team […] I think we complement each other. That’s the biggest facilitator, the fact that we work as a team and support each other and complement each other.*

*(P5; physician): It is a facilitator, a way in which palliative teams are designed with their pluridisciplinarity and their attention focused on the needs of the patient and family, which means that we can be alert to spiritual needs and can act as a team, each giving their contribution.*

Participants also highlighted the facilitating role of congruent spiritual care, which implies the ability to execute a certain task or action with the necessary knowledge, values, and attitudes. This understanding resulted in increased reverence for patients/families and promoted the development of intentional and trustworthy connections that are essential for providing effective spiritual care.

*(P1; physician): I think it is extremely important that we know how to get to the heart of the matter and have the competence to “touch” spiritual issues that may be sensitive to the patient without causing harm to the patient.*

*(P7; social worker): We are there to facilitate spiritual care according to what the patient considers to be best for them.*

*(P6; psychologist): I think there has to be an effective combination of our academic, human, and moral training and our level of sensitivity and ability to surrender to others.*

*(P15; nurse): If the person feels comfortable addressing this issue, we strive to fulfill their wishes or desires. Above all, we are facilitators, striving to adjust spiritual care to each person’s needs.*

Some participants reinforced the importance of practices that promote spirituality, such as reading and practicing prayers, praying the rosary, and celebrating religious ceremonies.

*(P1; physician): For many patients, praying the rosary is a very important activity.*

*(P5; physician): We already had the example of a lady who went to the sanctuary of Fátima to attend mass; she went with her family (she used the magic ambulance project, which aims to fulfill significant desires). I remember that the lady that day was even more interactive than usual. She returned to the unit the same day and the following day she died. We believe that that trip was calming for her and her family and allowed her to leave in peace.*

*(P9; nurse): Promoting activities that already happened before in their daily lives, such as praying the rosary and receiving communion, allows them to express their spirituality and talk about it openly.*

*(P14; nurse): I remember, for example, the permanence of significant objects, such as the rosary or a saint on the bedside table, which were important to some people. And, by allowing these objects and symbols, patients became more comfortable.*

At the same time, participants believed that the establishment of a therapeutic alliance facilitates meeting the spiritual needs of those who suffer, whereby each party is potentially transformed by the other in the context of a healing relationship. During periods of chaos, patients find comfort in the conversations and support offered for their spiritual well-being, in the company during their spiritual trajectory. While professionals often experience apprehension when starting and participating in conversations regarding spirituality, patients can find healing amidst their suffering in the presence of a compassionate professional.

*(P5; physician): While it is difficult to talk openly about spirituality, I try to understand what is bothering the patient; I try to provide an environment of trust so that the patient can explain to me what is happening and what they feel.*

*(P6; psychologist): Therefore, in this process of active listening and within the spirit of mission, one should listen to the other person’s life story and try to help them in accordance with their personality and their life goals at that moment. The therapeutic relationship is of paramount importance in the support of healing.*

*(P7; social worker): Empathy towards others is fundamental. […] In other words, as a professional, you have to be aware that this could be important for that person. And when that happens, your empathy toward others, your way of being, and your sensibility increase.*

Two participants added that, within the scope of spiritual conversations, patients with greater personal resources are better able to cope with their illness and alleviate their suffering.

*(P9; nurse): Sometimes when nothing else works in terms of medical intervention, patients will seek strength in faith, allowing them to better accept their situation.*

*(P6; psychologist): Patients often say “I’m prepared to die, I’m prepared to leap” […] and they find inner peace in their spirituality and self-knowledge.*

#### 3.2.3. Strategies for Competent and Spiritual-Centered Care

As spiritual care is an intrinsic part of palliative care, three main implementation strategies were mentioned: (1) assessment focused on the spiritual needs of an ill person; (2) implementation of effective spiritual accompaniment; and (3) development of self-knowledge and interpersonal relationships in work environments.

Throughout the illness trajectory, people with palliative needs face multiple challenges in the search for the meaning of life. Therefore, participants emphasized the need for an assessment focused on the spiritual needs of the ill person.

*(P1; physician): Almost always, we end up asking if patients have any beliefs, any religious practices, any spiritual needs. This assessment is essential to get closer to their particularities.*

*(P2; nurse): When there is an opportunity, we try to talk about spiritual practices, whether you are a believer or not, whether you believe in a superior being […] and then see how we can help.*

*(P6; psychologist): In my clinical practice, I try to understand if people are calm in their spirituality or if they need help finding inner peace.*

Several participants also highlighted the importance of effective spiritual accompaniment, which assumes that spirituality and sacredness are part of our lives and that we can become more sensitive to their presence through reflection, engagement, and prayer.

*(P3; nurse): Spiritual support helps people accept death, being with them, supporting them. Our presence, being with the person, and asking them what they need to do to feel at peace, are fundamental elements in spiritual care.*

*(P13; physician): Accompanying means talking openly about spirituality, about meanings […] but it is above all being there, listening, and not judging. […] There may be aspects that do not make sense to us as spiritual beings. However, being there and making an effort in this process is the most important thing.*

Lastly, participants pointed out the need for the development of self-knowledge and interpersonal relationships in work environments. Spiritual self-awareness and spiritual competency are developed through reflective practices, training programs, and team debriefing sessions.

*(P1; physician): In-service training using roleplay and practical exercises helps a lot in communicating with patients and families. Having moments of self-knowledge promoted as a team helps us deal with complex patients, and this increases our competency.*

*(P6; psychologist): Spiritual retreats awaken us to spiritual well-being but also help our patients. After all, if we are not well and do not take care of ourselves, the helping relationship is compromised.*

## 4. Discussion

The purpose of this study was to qualitatively explore the perceptions and practices of palliative care professionals concerning spiritual care. The thematic analysis of the findings resulted in three main themes and eight subthemes. The first theme reports the meanings attributed to spirituality and spiritual care; the second theme identifies a range of barriers and enablers affecting spiritual care; and the last theme presents various strategies that professionals recognize can promote spiritual-centered care.

The findings obtained indicate a consensus among participants regarding the fact that spirituality constitutes a universal human dimension and an integral element of palliative and EoL care [36,47,51]. As Balfour Mount [52] states, human beings are inherently intrinsically spiritual, since all people are in a relationship with themselves, with others, nature, purpose, or the sacred. However, professionals in general, and even in palliative care, are interested in incorporating spirituality into their roles. This interest is often attributed to scientific studies that identify a link between spirituality and well-being, and spiritual care’s positive impact on mental and physical health [36,53].

Spirituality and religion are distinct in their scope, but there was no clear distinction between both concepts across the participants’ narratives. While religion is communal and associated with particular rituals, institutional affiliations, and social connections, spirituality revolves around personal encounters with the intangible and the acknowledgment of forces beyond our own existence [54]. According to Bożek et al. [55], religion is mostly seen as a societal occurrence, whereas spirituality is often individual and within a specific context. Spirituality is considered a universal phenomenon, whereas religion is typically understood as how people might express their spirituality. Therefore, the sacred is not restricted to religion, as traditionally thought, but can manifest in secular settings such as nature, national gatherings, sporting events, and social justice initiatives [56]. Although spirituality and religiosity share comparable origins connected to transcendence, they should not be used interchangeably. Despite certain commonalities in their significance, they are distinct.

Although palliative care professionals tend to resist addressing spiritual issues with their patients, the provision of spiritual care in a culturally intertwined and pluralistic world demands an ontological basis [17]. Although spiritual care is not easy to operationalize, it allows more explicit and inclusive reflections about the individual and will help health professionals assess perspectives and the extent of a patient’s construction of existential meaning, as well as the existing sources of suffering and spiritual distress [17,57].

Our findings suggest that being near death makes experiences of spiritual awakening a common condition in PC. These experiences often “evoke an ineffable sense of deep inner knowing, understanding, ‘remembering,’ or ‘unveiling’ of one’s true nature, as well as experiences of peace and equanimity, bliss, ecstasy and aliveness, feelings of awe, sacredness, gratitude and reverence, and of abundant, unconditional love” [58] (p. 2). Several authors claim that the experience of proximity to death can, in itself, create concentrated opportunities for spiritual growth, which, among other things, includes feelings of connection and peace [59,60].

Another meaning attributed to spiritual care by our participants focuses on relational spirituality, the individual and subjective assessment of spirituality, which is expressed through social connections, especially in therapeutic relationships. Spirituality “has increasingly been conceptualized in relational terms, and the term ‘relational spirituality’ has been frequently employed in recent conceptual and empirical literature” [61] (p. 1).

Like other studies regarding barriers to the implementation of effective spiritual care, our participants reported organizational variables, such as an inadequate professional-to-patient ratio, lack of time, high workload, and late referral of patients [46,62,63]. Other factors of a personal nature (such as the lack of specialized training in spirituality, the lack of professionals’ self-knowledge and self-awareness) as well as social factors (such as multiculturalism and taboos of patients and families regarding death) were referred to as limitations to effective spiritual care. Several studies conclude that healthcare professionals do not feel ready or sufficiently educated and lack the confidence and competence to address spiritual issues with patients [23,37] or feel that others could perform the role better than them [64]. Although most professionals report that they experience difficulties or barriers, mainly due to the lack of specific training, they all tend to provide this type of care, with a greater or lesser degree of competence. Through an inter-religious/spiritual stance, spiritual support in palliative care is becoming a huge challenge that has been demonstrated cross-culturally [10,65,66].

In contrast, participants identified a pool of factors that facilitate spiritual care for palliative patients and their families, including interprofessional collaborative practice, congruent spiritual care, and therapeutic alliance. An interprofessional approach to PC involves professionals from various areas, including doctors, nurses, social workers, psychologists, and chaplains and/or spiritual assistants. This breadth of professionals allows for a more comprehensive and sensitive understanding of spiritual needs, promoting a more compassionate and integrated care environment. It is up to professionals to identify these needs, what they can address themselves, and where they need collaboration or referral from colleagues from other disciplines [67,68]. Puchalski et al. [2], in a consensus conference on the quality of spiritual care in PC, state that spiritual care must be collaborative and provided by qualified interdisciplinary professionals. Likewise, PC professionals need to take time for their patients’ spiritual needs, remain open-minded, build a relationship of trust and compassion, and respect their limitations [11] to provide congruent and authentic spiritual care. Establishing a sense of trust and rapport between patients/caregivers and the healthcare team is an essential aspect of spiritual care, and one effective method involves attentively and actively listening to their concerns [69,70]. Using a spiritual-centered care approach, the resources of patients and families should also be integrated during the provision of spiritual care plans fostering dignity and the relief of suffering [47,71]. Our findings indicate that participants believe that spiritual care influences a patient’s health in some way, positively relating it to coping with a serious illness [72]. Our study strongly indicates that care providers must sustain the ethos of spiritual care, which involves facilitating spiritual practices and making the necessary adjustments to the environment, such as creating a sacred space. This may include arranging the layout and design of rooms and incorporating symbolic objects, natural elements, and artwork [73].

Our findings echo previous studies regarding strategies used by professionals to promote spiritual care in PC contexts. Professionals should be better equipped to create a spiritual care treatment plan that explicitly reflects the “patient’s ontological grounding” [17]. For this purpose, Benito et al. [74] suggest assessing the three dimensions of spirituality, namely, the intrapersonal (i.e., relationship with oneself), interpersonal (i.e., relationships with others), and transpersonal (i.e., awareness of transcendence). Rumbold highlights the significance of mutuality in spiritual caring [75], when both the patient and caregiver acknowledge one other’s humanity and actively participate in co-creation. The focus of cocreating efforts is developing a care plan involving patients, family members, and healthcare providers [76].

Lastly, continuous recognition and emphasis on spiritual care are necessary for both education and patient care. The significance of spiritual matters must be consistently reaffirmed, and clinical practice should provide opportunities for dedicating time to spiritual caring [46,77,78,79]. Professionals are aware of their lack of knowledge, understanding, and skills, and desire to improve their competencies [78,79]. Therefore, they need to develop self-knowledge, interpersonal relationships in the work environment, and skills that help them deliver spiritual support and aid the individuals involved in formulating an optimal spiritual care treatment plan [17]. According to Hvidt et al. [80], the basic nature of spirituality and its connection to human choices and beliefs makes the delivery of spiritual care essential to healthcare, yet also challenging. Although it may not be a simple task, the authors concur and propose that spiritual care competency should be considered an essential skill that both individuals and healthcare professionals should cultivate throughout their lifetimes [79]. Interestingly, palliative care practitioners who are highly skilled in providing spiritual care and have a strong personal spirituality are less likely to experience staff burnout [36,81].

### 4.1. Study Strengths and Limitations

This qualitative inquiry provides a well-timed analysis of the spiritual care challenges and opportunities faced by PC professionals and might help stakeholders make informed suggestions on providing compassionate spiritual practices. The research was transparent and rigorous during data collection and analysis by utilizing a reflective research journal and conducting peer reviews of the established themes and sub-themes.

Despite its strengths, this exploratory investigation has several limitations, one of which is the limited generalizability of our findings due to the small size of our sample, extracted from a single institution. Therefore, further research using a larger and more varied sample is necessary to validate our findings. Second, the use of convenience sampling in this study may have led to a sample bias, namely an overrepresentation of participants who are spiritually minded and driven. Third, there was a lack of data source triangulation to gather diverse perspectives, including those of researchers, care providers, and patients/families. Further research is needed to investigate all those involved in spiritual care to better grasp what needs to be done to address their needs and foster customized interventions. Fourth, we did not separate the perspectives of different professionals during content analysis but treated them as a whole. Finally, this study outlined one way to investigate spiritual care in the Portuguese context, deeply rooted in Catholicism. It made no mention of the structural reasons or the plethora of other systemic problems (e.g., hegemonic ideologies associated with social inequalities, globalization, heteropatriarchy, and secularism) that may have limited spiritual-centered care [82]. This might potentially restrict the transferability of our findings to different situations. It is important to consider these structural and systemic features while doing further research utilizing a mixed-methods approach.

### 4.2. Implications for Practice

Spiritual care facilitates the integration of the significance, psychological representations, and values associated with spiritual and/or religious beliefs and symbols [53]. Narratives play a crucial role in giving patients’ lives significance and may be understood and approached from many therapeutic viewpoints; therefore, they are essential for a comprehensive treatment [80]. The concept of “narrative care” has shown that empathetic listening has therapeutic advantages. When used in spiritual care, it enables professionals to best understand a patient’s “total pain” [82] and to work with hope, positive revaluation, the art of dying, and rituals [16,83,84]. While spiritual care might progress through sequential phases [17], individuals may respond idiosyncratically in each stage. Seeing spiritual care as a process implies that institutions should develop a well-defined conceptual framework integrating spiritual care within everyday work and routines [17]. The looming challenge in palliative care is promoting conditions where a patient can develop a sense of ultimate meaning and make meaning of their situation [20].

Self-knowledge is also a crucial element in developing competence and cultivating spiritual awareness. Professionals must examine their personal beliefs and attitudes towards various spiritual values related to death and dying, particularly while caring for palliative patients. This is crucial since regularly seeing death forces individuals to confront their own mortality.

Evidence supports that neglect of spiritual care practices is related to insufficient education and training [30,63,85,86,87]. Professionals can enhance their ability to deliver spiritual care more effectively by undergoing continuing education and training. This would not only provide a deeper comprehension of the fundamental concepts of spiritual care but also help to develop skills on how to use this knowledge in their clinical work. Likewise, it is imperative to provide a pragmatic roadmap for identifying spiritual distress and collaborating with colleagues to guarantee that patients receive spiritual care aligned with their beliefs and values [88]. Training programs that enhance spiritual care competency, through education and self-reflection, are necessary to appropriately prepare professionals [89,90]. Furthermore, it is widely acknowledged that educating practitioners in moral reasoning can facilitate the advancement of ethical and fair decision-making in the context of healthcare for patients from diverse cultural backgrounds.

Healthcare institutions and ongoing educational programs need to incorporate structured training on effectively addressing communication and spiritual matters into their curriculum. Indeed, Syihabuddin [91] and Song [92] mentioned there is a need for pedagogical practices oriented toward spiritual values, known as transformative spiritual pedagogy. This pedagogical approach fosters students’ spiritual attitudes, cultural identity, morality, proper social behaviors, well-being, and functionality [93]. One valid educational tool that may help students be with someone who suffers and address their spiritual needs proposes five attitudes: “Prepare (P), Ask (A), Listen (L), Validate (V), and consult an Expert (E)” [94] (p. 1215).

Finally, marginalized communities often rely “on spirituality to counter the spiritual injury compounded by sexism, racism, ableism, ageism, and classism” [95] (p. 3). In this sense, professionals need to foster a purpose for living, inner strength, and meaning among the most vulnerable, thus contributing to the democratization of society and accelerating progress towards the Sustainable Development Goals [96].

## 5. Conclusions

As far as we know, this is one of the first attempts carried out in Portugal to explore the perceptions and practices of palliative care professionals concerning spiritual care. Three significant themes were derived from thematic analysis: (1) spiritual care as key to palliative care, (2) floating between “shadows” and “light” in providing spiritual care, and (3) strategies for competent and spiritual-centered care. Our findings state that spirituality is an idiosyncratic characteristic. A life-threatening condition can challenge a patient’s perception of their existence, as they are compelled to face their mortality. This may give rise to profound philosophical inquiries that, if unresolved, might trigger an existential crisis. Often, during this trajectory, spirituality is awakened and people are more aware of interpersonal spirituality, namely in relationships with others, especially therapeutic relationships. Spiritual care faces several challenges, including organizational variables (i.e., late referral to palliative care, shortage of staff, lack of standards in professional to-patient ratio, lack of time, and high workload), as well as lack of specialized training in spiritual care and deficits in spiritual self-awareness of professionals.

To overcome barriers to spiritual care, it is necessary to enhance interprofessional communication. We also recommend that spiritual care should be adequately valued, prompting staff to guarantee its accessibility for all patients and caregivers. Inclusivity and diversity emphasize the need for spiritual carers to acknowledge the choices of individual patients and carers, particularly concerning the cultural components of spiritual care. It also underscores the significance of care providers attending to their own spiritual knowledge and worldviews. Patients and their caregivers must be prioritized by acknowledging their personal preferences and satisfying the organizational imperative of upholding human dignity. To provide spiritual care, institutions must acknowledge the need for such care and offer staff training and assistance. Spiritual care helps both patients and staff, as it enables them to provide improved support to patients.

## Figures and Tables

**Table 1:** Participants’ background.

Participants	Age (Years)	Sex	Religious Affiliation	Professional Category	Professional Experience (Years)	Years of Work in Palliative Care	Training in Spiritual Care
P1	34	Female	Catholic	Physician	8	1	No
P2	50	Female	Catholic	Nurse	29	2	No
P3	38	Female	None	Nurse	16	2	No
P4	46	Female	Catholic	Nurse	22	2	No
P5	36	Female	None	Physician	10	4	No
P6	47	Female	Catholic	Psychologist	18	13	Yes
P7	36	Female	None	Social worker	8	3	No
P8	30	Female	Catholic	Nurse	8	2	Yes
P9	37	Female	Catholic	Nurse	13	2	No
P10	37	Female	Catholic	Nurse	8	2	No
P11	33	Female	Catholic	Nurse	5	2	No
P12	36	Female	Catholic	Social worker	10	6	No
P13	37	Female	Catholic	Physician	12	6	No
P14	36	Male	Catholic	Nurse	14	1	No
P15	45	Female	Catholic	Nurse	23	2	No

## Data Availability

The data are available upon reasonable request. This article is based on the first author’s master’s dissertation in Palliative Care at the School of Health Sciences—Polytechnic University of Leiria.
